# HS-SPME-MS-Enose Coupled with Chemometrics as an Analytical Decision Maker to Predict In-Cup Coffee Sensory Quality in Routine Controls: Possibilities and Limits

**DOI:** 10.3390/molecules24244515

**Published:** 2019-12-10

**Authors:** Erica Liberto, Davide Bressanello, Giulia Strocchi, Chiara Cordero, Manuela Rosanna Ruosi, Gloria Pellegrino, Carlo Bicchi, Barbara Sgorbini

**Affiliations:** 1 Dipartimento di Scienza e Tecnologia del Farmaco, Università degli Studi di Torino, 10125 Turin, Italy; davide.bressanello@unito.it (D.B.); giulia.strocchi@unito.it (G.S.); chiara.cordero@unito.it (C.C.); carlo.bicchi@unito.it (C.B.); barbara.sgorbini@unito.it (B.S.); 2 Luigi Lavazza S.p.A, Strada Settimo 410, 10156 Turin, Italy; Manuela.Ruosi@lavazza.com (M.R.R.); Gloria.Pellegrino@lavazza.com (G.P.)

**Keywords:** HS-SPME-MS-enose, coffee, prediction of in-cup sensory quality, chemometrics

## Abstract

The quality assessment of the green coffee that you will go to buy cannot be disregarded from a sensory evaluation, although this practice is time consuming and requires a trained professional panel. This study aims to investigate both the potential and the limits of the direct headspace solid phase microextraction, mass spectrometry electronic nose technique (HS-SPME-MS or MS-EN) combined with chemometrics for use as an objective, diagnostic and high-throughput technique to be used as an analytical decision maker to predict the in-cup coffee sensory quality of incoming raw beans. The challenge of this study lies in the ability of the analytical approach to predict the sensory qualities of very different coffee types, as is usual in industry for the qualification and selection of incoming coffees. Coffees have been analysed using HS-SPME-MS and sensory analyses. The mass spectral fingerprints (MS-EN data) obtained were elaborated using: (i) unsupervised principal component analysis (PCA); (ii) supervised partial least square discriminant analysis (PLS-DA) to select the ions that are most related to the sensory notes investigated; and (iii) cross-validated partial least square regression (PLS), to predict the sensory attribute in new samples. The regression models were built with a training set of 150 coffee samples and an external test set of 34. The most reliable results were obtained with acid, bitter, spicy and aromatic intensity attributes. The mean error in the sensory-score predictions on the test set with the available data always fell within a limit of ±2. The results show that the combination of HS-SPME-MS fingerprints and chemometrics is an effective approach that can be used as a Total Analysis System (TAS) for the high-throughput definition of in-cup coffee sensory quality. Limitations in the method are found in the compromises that are accepted when applying a screening method, as opposed to human evaluation, in the sensory assessment of incoming raw material. The cost-benefit relationship of this and other screening instrumental approaches must be considered and weighed against the advantages of the potency of human response which could thus be better exploited in modulating blends for sensory experiences outside routine.

## 1. Introduction

Coffee is universally considered a comfort food and is widely consumed because of its particular flavour. The flavour of coffee is the result of the transformations of the harvested bean to the final roasted product. The chemical composition of coffee is variable, meaning that its sensory profile can radically change according to species, origin, year of harvest and post-harvest treatment. Roasters therefore need to constantly control the quality of their incoming beans.

Coffee is evaluated by its visual appearance; colour, bean uniformity, shape and size, number of “defective beans” and taste. However, coffee beans may have a pleasant aspect, but present an unpleasant taste because of contamination and chemical modification that may have occurred during storage, processing and transport from origin to the roaster’s warehouse. Tasting, of course, plays a fundamental role in coffee quality evaluation, meaning that “cupping” is routinely used [1,2,3], to evaluate a lot (or a crop) for blend formulation, or “single origin” coffee and ultimately also to determine its price [4]. Nevertheless, cup tasting is time-consuming as it requires a specialised panel, who must be trained and aligned. Furthermore, the present trend in the food industry is to move panels from routine to the development of new finished products with given or peculiar flavour characteristics.

Flavour can be considered the signature of a product [5,6,7,8], and defining a relationship between chemical profile and aroma sensory impact is an important challenge for both the analytical and industrial fields, as they aim to achieve an objective and fast routine evaluation of a product with an automatic analytical procedure [9,10,11,12,13,14].

The use of rapid techniques in coffee analysis is constantly increasing. For instance, NIRS has been used to discriminate between coffee species and blends [15], to define the roasting degree of coffee beans and to quantify several bioactive coffee compounds, such as caffeine, trigonelline and chlorogenic acids and to predict sensory attributes, such as acidity, body, bitterness and the quality of espresso coffee [16,17]. Proton transfer reaction mass spectrometry (PTR-MS) and Laser Ionisation Mass Spectrometry (REMPI/TOFMS) have been used on-line, coupled to a Probat roaster, to control the roasting process, from volatile formation, and to study the kinetics of flavour development [18,19,20,21,22,23].

E-nose technology based on an array of electronic chemical sensors has also been attractive for industry. E-noses have been used in coffee research to differentiate Robusta from Arabica beans and to discriminate aromas via a fine-tuning process that involves altering the sensor materials [24]. The main advantages of these technologies are their cost-effectiveness, the fact that they can be easily integrated into a productive process, and the rapidity with which results can be obtained compared to traditional chemical and chromatographic methods. Despite these features, only few applications in industry have been described for these techniques, mainly because of the relatively low robustness, selectivity and reproducibility of the sensors, the large amount of data required to calibrate instrumentation and the resulting need for complex data analysis and algorithms [25,26,27].

These limits can be overcome by non-separative MS methods, better known as mass spectrometry-based electronic noses or MS-EN, which, when combined with headspace sampling, provides a representative, diagnostic and generalised mass spectrometric fingerprint of the volatile fraction of a sample, without prior chromatographic separation. With this approach, each *m*/*z* ratio acts as a “sensor” whose intensity derives from the contribution of each compound that produces that fragment. It was introduced by Marsili in 1999 [28] to study off-flavours in milk and, since then, has been applied in the quality control of herbs and spices, in the authentication of food, to classify defective products, and to predict the sensory properties of food [13,29,30,31,32,33].

The present study applies this method to the coffee headspace, as sampled by HS-SPME, to develop an instrumental prediction model as an analytical decision maker for routine controls to define in-cup sensory quality in accepting incoming samples [10,12,14,34]. Coffee samples underwent sensory evaluation via monadic profiling, according to SCA [35] protocols, and were analysed using HS-SPME-MS in combination with multivariate statistical analysis.

## 2. Results and Discussion

### 2.1. Sensory Analysis

Sensory data show that the scores for aroma properties were spread over the full range (scale 0–10), although the highest values were poorly represented, as expected, because of the intrinsic characteristics of the samples, such as species, origin, primary processing, and the life-span of the study. The standard deviations (SD) of the attributes (Table 1) are very low considering the high numbers of both of the samples investigated (184) and judges (6). A high Coefficient of Variation (CV) was observed for some attributes, fruity, flowery and spicy, meaning that these sensory properties were rated as very high or very low by the panel. CV relates the SD to the mean values of the aroma properties and provides a more representative evaluation of the importance of SD. In addition, it is a useful measure for comparing the dispersions of two or more attributes measured on different scales.

**Figure 1:**
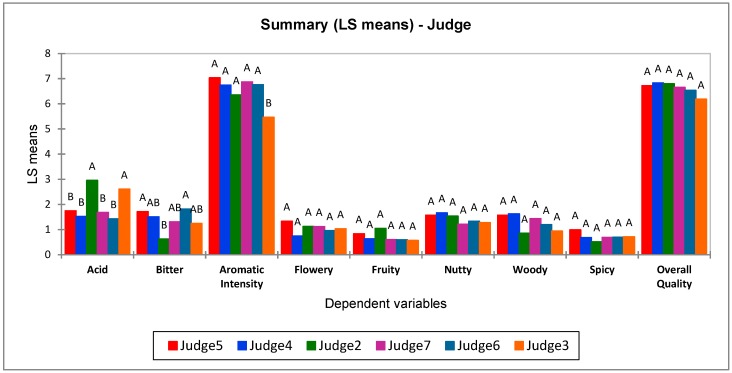
ANOVA and post-hoc Tukey’s test results on the ability of the judges to rate the different attributes. The same letter means that the judges involved rate the attributes in the same way at a confidence level of 95%.

ANOVA analyses and a post-hoc Tukey’s test provided information on the judges’ ability to evaluate the sensory attributes. Figure 1 shows that judge 3 does not perform similarly to the others for the aromatic intensity coffee property, and, together with judge 2, for acid notes, while judge 2 has a different evaluation for bitter attribute compared to the others. All judges are, however, aligned in rating the scores of the other attributes. These two judges were therefore not taken into consideration for the attributes in which their variance was not comparable to the others. The score averages were used as the “main scores” for the nine attributes in the following elaborations.

### 2.2. How a TAS System Based on the MS-Enose Works

**Figure 2:**
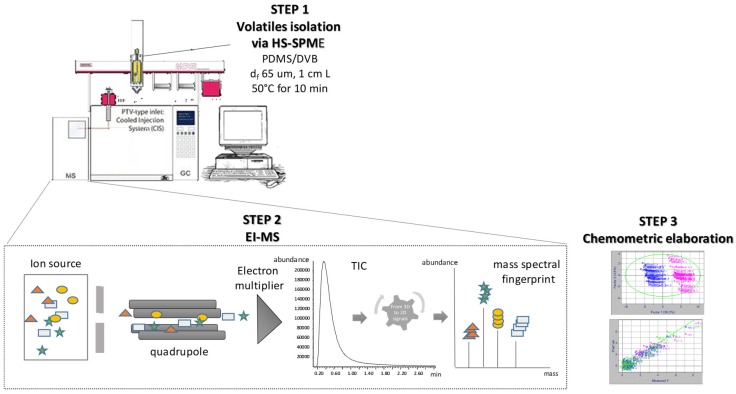
Schematic representation of the Total Analysis System (TAS) system used.

The platform adopted allowed experiments to be run with an high throughput Total Analysis System (TAS) [36], which consisted of an autosampler for fully automated HS-SPME sample preparation on-line, and was directly combined to a mass spectrometer (MS), through a void column that was thermostatted in a GC oven, whose output signal (data) was elaborated on-line and then processed using chemometric software. The HS-SPME-MS TIC (Total ion Current) pattern is a single peak whose mass spectrum is representative of the fingerprints of the whole coffee volatile fraction (Figure 2).

The corresponding mass spectral fingerprint is highly reproducible and ideally suited for further chemometric elaboration as it only consists of whole masses [37,38].

**Figure 3:**
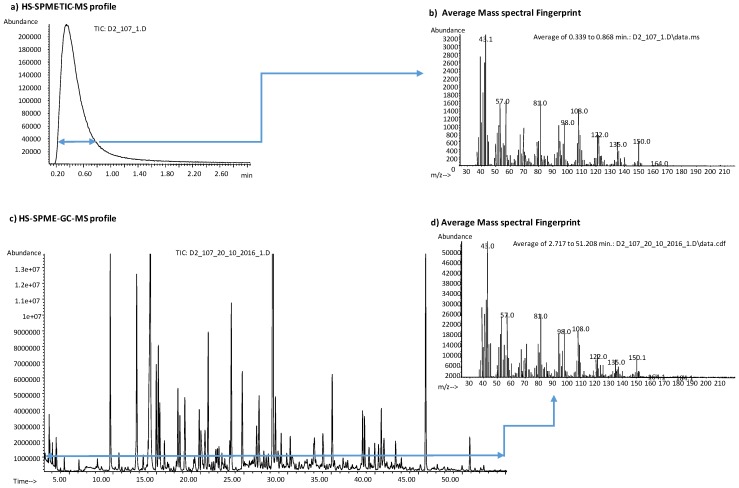
Analytical output signals of an Arabica roasted coffee sample from: (**a**) HS-SPME-MS-enose profile; (**b**) average HS-SPME-MS-enose mass spectral fingerprint that corresponds to the TIC data from MS-enose; (**c**) HS-SPME-GC-MS chromatogram; (**d**) average HS-SPME-GC-MS mass spectral fingerprint of the whole chromatographic profile.

Compared to conventional GC-MS, mass spectral fingerprints provide total information about each sample and may even be more helpful and meaningful, in routine control screening, than the characterisation of each individual component in that sample. The reliability of the mass spectral fingerprint is demonstrated by the comparison of the high degree of overlap between the average mass profile of an Arabica volatile fraction obtained using the non-separative techniques (HS-SPME-MS-enose) and the average total spectrum over the total analysis time from a conventional HS-SPME-GC-MS analysis, as reported in Figure 3.

### 2.3. Signal Processing and Chemometric Workflow

The mass spectral fingerprint encompasses all the chemical information on the volatile fraction of an analysed sample, while diagnostic and informative fragments can be correlated to a compound or a class of compounds. The mass fingerprint is displayed on a plot that reports the mass fragments (*m*/*z*) within the selected mass range on the *X*-axis, and the ion abundances for the mass fragments on the *Y*-axis (Figure 3).

**Figure 4:**
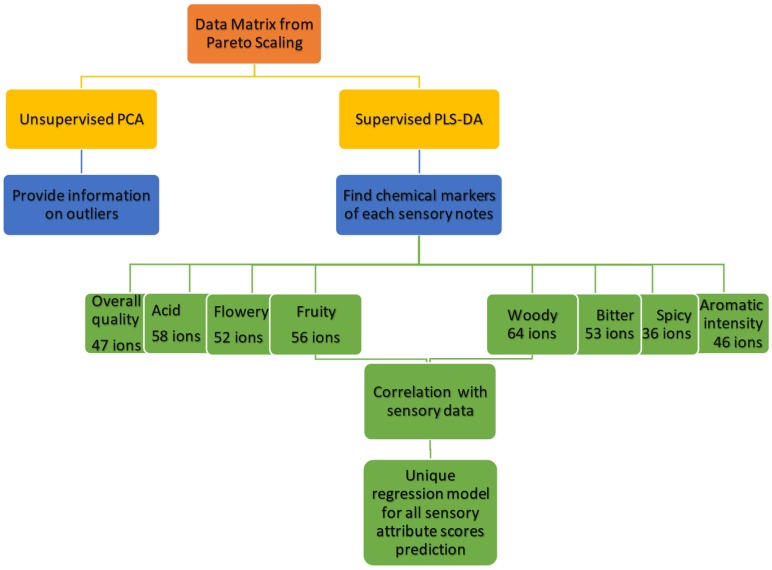
Workflow of the chemical data processing used to obtain the regression model.

The use of chemometrics to extract the significant and useful information from the complex data matrix, however, requires the profile to be precise, in particular when data monitoring is carried out over a long period and when a mathematical model for classification or correlation has to be generated. The chemometric tools adopted in this study were, in sequence: (i) Principal Component Analysis (PCA) to identify outliers; (ii) Partial Least Square Discriminant Analysis (PLS-DA) carried out on the sensory scaled samples (low-high score range) to identify the fragment ions that are most closely related to each sensory attribute; and (iii) Partial Least Square Regression (PLS) to correlate chemicals to sensory attributes, and to evaluate the ability of extracted chemical variables to predict sensory scores. The data processing work-flow is reported in Figure 4.

The consistency of the SPME fibres over time was ensured by testing six fibres of the same lot with a text mixture, and selecting those whose responses could not be distinguished using ANOVA; their performance was periodically monitored using the same test mixture. Before chemometric processing, the matrix was cleaned of ground fragments that may have interfered with data elaboration, e.g., the fragments at *m*/*z* = 44 (CO_2_), *m*/*z* = 73, 133, 147 and 177 (system bleeding), and *m*/*z* = 149 (derived from phthalates). The resulting data matrix was than subjected to internal normalisation vs. the most abundant ion (*m*/*z* 43) and standardisation using Pareto scaling [39,40].

The HS-SPME-MS pattern is informative because the intensity of each ion (*m*/*z*) derives from the contribution of all components that present that fragment in their ionisation pattern. Chemometric elaboration consists of a series of steps to “extract” significant information from the MS fingerprint for further sensory score prediction.

The data matrix from the Pareto scaling was first submitted to an unsupervised exploratory investigation using principal component analysis (PCA) to detect sample outliers. The samples were found to be homogeneously distributed along the first three PCs with a cumulative explained variance of 83.15%, and the data indicated two populations of samples on the first PC that were related to the coffee species (Figure S1 in Supplementary Material). The fragment ions that derived from the volatile fraction therefore provided information on the chemical diversity of the investigated set of samples.

A supervised Partial Least Square Discriminant Analysis (PLS-DA) was then applied to the reprocessed MS spectral fingerprints on selections of samples that had the highest and the lowest scores for each sensory attribute in order to extract ions that had a high impact on sample discrimination (low vs. high scores). A cross-validation (CV = 5) was set to run the PLS-DA. The variable importance for projection (VIPs) scores estimate the importance of each variable in the projection that was used in a PLS-DA model, and is often used to select variables. VIPs higher than 1 and with a low standard deviation were considered for the extraction of the relevant ions that described each sensory attribute.

**Figure 5:**
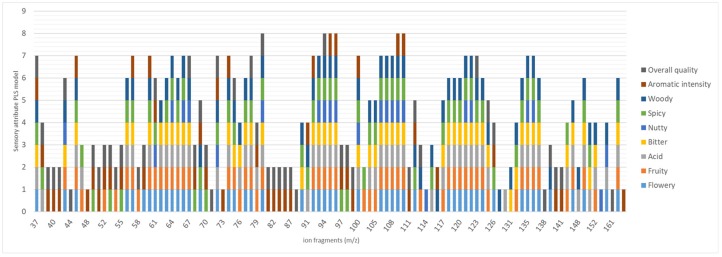
The importance and occurrence of each selected mass fragment (*m*/*z*) in the partial least square (PLS) regression model of every sensory attribute.

Table 2 shows the significant ions that were selected for each sensory attribute under investigation, together with their VIP value and standard deviation. Results show that the total number of relevant ions is different for each considered sensory attribute and, in particular, that sensory qualities share several ions Table 2 and Figure 5.

A PLS model was built for each single sensory attribute to verify the relationship between the chemical ion fragments (*m*/*z*) (i.e., the chemical components of the volatile fraction) and the sensory profile, and to predict the sensory scores. Figure 5 shows that the ions selected from the PLS-DA and used to design the corresponding regression model for each sensory feature are involved in more than one feature.

For example, the typical base fragment of organic acids *m*/*z* 60 in coffee (the most abundant of them being acetic acid and 3-methyl butanoic acid), is depicted in 7/9 regression models, while *m*/*z* 150/152, predominantly related to methoxy phenols, are only present in 4/9 regression models. This result is in agreement with results reported in Ribeiro et al., who underlined the importance of the co-participation of several volatiles in describing various sensory features in fifty three Arabica coffees [41].

Moreover, the mass spectral fingerprint provides some information on the volatiles that characterise the samples. For example, *m*/*z* 108 is mainly related to several alkylated pyrazines and pyrrole derivatives, *m*/*z* 95/96 are associated to furfuryl products, and 135/137/150/152 are primarily related to methyl-ethyl pyrazine and methoxy phenols.

**Figure 6:**
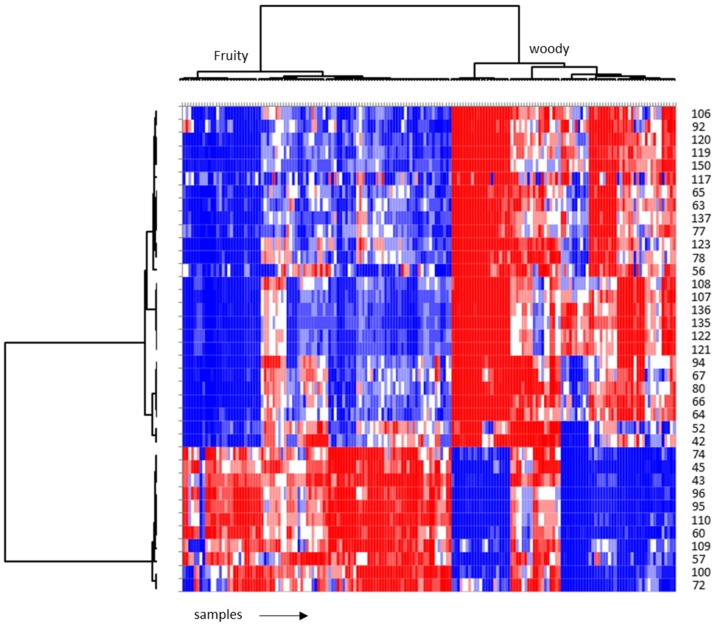
Heat-map of a group of the selected samples that present woody and fruity features.

The ability of the VIP-selected ions to describe the sensory characteristics of samples can be visualised for the most fruity and woody samples in the heat-map in Figure 6, which shows a clear discrimination between the samples with these two sensory profiles thanks to mass fingerprinting. The rows indicate the *m*/*z* ions, and the columns the investigated samples. The colour scale varies from blue (low abundance) to red (high abundance). A hierarchical cluster analysis (HCA) of both the rows and columns shows that volatile distributions differ according to their normalised response across samples. Figure 6 highlights that the ion-intensity ratios across samples in place of the different quality volatiles is very effective to discriminate the two cluster of samples linked to their sensory peculiarity [3,42,43].

The ions selected using PLS-DA were then used as independent variables to evaluate the relationship with sensory data and the ability to predict scores (dependent variables) of each sensory attribute by developing a specific and optimised regression model for each feature. All sensory notes have been modelled through a PLS algorithm; the evolution of each sensory note over the sample sets has been related a different number of variables (Table 2). The flexibility of the prediction model for each sensory attribute was evaluated in samples that covered a range of seasonality, origins and crops and then tested the models with an external test set (Table 3). Acid, Bitter, Spicy and (to a lesser extent) aromatic intensity and flowery PLS prediction models show good performance. The R2 values indicate that nearly more than 50% of the variance in the measured scores is explained by the models (i.e., the selected mass spectral fragments used to describe the model). This is quite a good result in consideration of the high variability of the training set. The goodness of the predictive capability is confirmed by the acceptable values of the root mean squared errors (RMSECV and RMSEP) reported for these attributes. The limit of acceptability for predicted values has been strictly fixed by the sensory panel, in ±1 score points. All RMSECV values are within or close to this interval while prediction on new samples show RMSEP slightly higher in particular for the overall quality and to a lesser extent for woody and nutty.

The models so far developed, however, still require nine different data elaborations. This is a serious limit for routine HS-SPME-MS-enose applications that can only be overcome with a unique multi-note sensory-score prediction model. The variable selection for the multi-note model was carried out by combining the matrices (ions fingerprints) used for the single-note prediction models. Fragment ions from the single-model note, without repetitions, were selected, the x matrix was then simplified and the number of variables reduced according to VIP values. This variable reduction was carried out to reduce the statistical noise, and maximise the information provided by each single note chemical fingerprint. According to the VIP values (VIP > 0.8), 78 ions out of 104 in the volatile fraction were retained in building the model, thus allowing the multidimensional structure of the prediction model to be simplified with negligible loss in performance.

**Figure 7:**
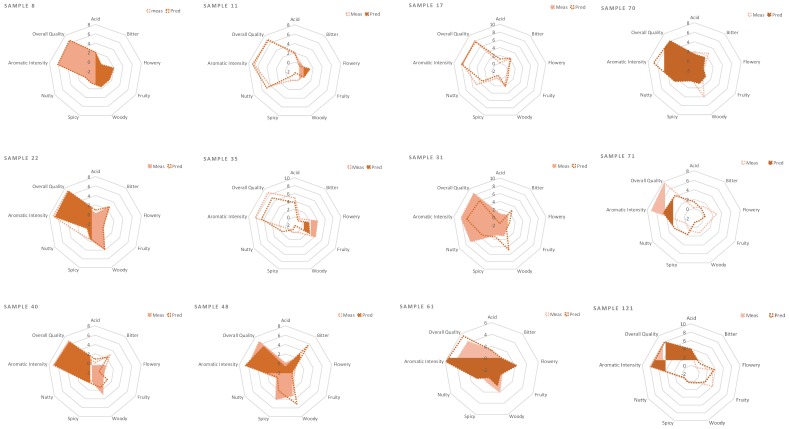
Comparison of the measured sensory profiles (from the panel) and predicted sensory profiles, from the developed model, of a selection of external test set samples. Sensory and chemical data were pre-processed using pareto scaling.

The regression model was built with a training set of 150 objects and an external test set of 34. The leave-p-out cross-validation method (*n* = 20) was used to select a suitable number of components from the PLS regression and to reduce the errors when the calibration model was used for the feature predictions of unknown samples. The results of the developed multi-note regression model for the prediction of sensory-attribute scores show that acid, bitter and spicy notes were the most reliable as they present a lower root mean square error in prediction (RMSEP). However, the mean error in the sensory-score prediction RMSEP in the external test set with these data fall within the range of ±2 (Table 3). The other attributes show discrete to good fitting between chemical and sensory data from the R2val values, and a better ability to predict the scores of the training and internal evaluation set, but fail to meet the expectation limit for acceptability given by the panellist (±1) when used to estimate new samples Table 3 and Figure 7. The high errors in prediction, in particular for samples with high sensory scores, probably occurred because of the unbalanced samples (i.e., the number of high scored samples was lower than that of the low scored ones for some attributes such as fruity, nutty, woody), making this part of the score range less well represented in the sample sets.

The low predictive ability for new set of samples may be due to: (a) the different species, origin seasonality and post-harvest treatments compared to other published work [23,41]; (b) the high noise caused by an unbalanced pool of samples for some attributes, such as nutty or flowery; (c) the difficulties linked to an excessively general lexicon to define the notes; and (d) compromises in the abilities of modelling for each sensory characteristic must be considered when building a multi-note model.

## 3. Materials and Methods

### 3.1. Samples

One hundred and eighty four coffee samples, with distinctive sensory notes, originating from a number of different countries were analysed. Coffee samples were kindly supplied by Lavazza S.p.A. (Turin, Italy). The roasting degree of each sample was carefully measured by ground bean light reflectance, with a single-beam Color Test 2 instrument Neuhaus Neotec (Genderkesee, Germany) at a wavelength of 900 nm, on 25–30 g of ground coffee. The roasting degree was set at 55° Nh, in order to be close to the international standardisation protocol for cupping [35]. Samples were roasted no more than 24 h before cupping and left at least 8 h to stabilise.

### 3.2. Descriptive Sensory Analysis of Coffee Aroma

The samples were submitted to sensory evaluation by a panel of six coffee experts. Aroma quality was assessed for a set of nine attributes, namely flowery, fruity, woody, nutty, spicy, acidity, bitterness, aroma intensity and overall quality. The quality and intensity of each attribute were simultaneously evaluated, on a scale from 0 to 10. ANOVA analysis with a post-hoc test were run to verify panel alignment on each attribute. Average scores from experts whose evaluations were similar were used as the “main scores” for the investigated attributes.

### 3.3. Head Space Solid Phase Micro Extraction Sampling

Volatiles were sampled using HS-SPME and an MPS-2 multipurpose sampler (Gerstel, Mulheim a/d Ruhr, Germany) which was integrated online with an Agilent 7890 GC coupled to a 5975 MS detector (Agilent, Little Falls, DE, USA). One point five grams of ground roasted coffee in a 20 mL vial were directly sampled by HS-SPME for 10 min at 50 °C at a stirring speed of 350 rpm. The SPME fibre was a PDMS/DVB df 65 μm, and 1 cm long (Supelco, Bellefonte, PA, USA). After sampling, the recovered analytes were thermally desorbed, by heating the fibre for 5 min at 250 °C, into the GC injector body, from where they were transferred on-line to the gas-chromatographic column. All samples were analysed in duplicate.

### 3.4. MS-eNose Instrument Set-Up

The GC oven and injector were maintained at 250 °C; injection mode, split; split ratio, 1/10; carrier gas, helium; flow rate, 0.4 mL/min; fibre desorption time and reconditioning, 3 min. The transfer column was uncoated deactivated fused silica tubing (dc = 0.10 mm, length = 6.70 m) from MEGA (Legnano, Italy).

MSD Conditions: ionisation, EI mode at 70 eV; temperatures: ion source: 230 °C, transfer line: 280 °C. Standard tuning was used and the scan range was set at *m*/*z* 35–350 with a scanning rate of 1.000 amu/s.

### 3.5. Data Acquisition and Elaboration

Data were acquired and processed using an Agilent MSD Chem Station ver. E.02.01.1177 (Agilent, Little Falls, DE, USA). Raw data were transformed using RapidDataInterpretation software by Gerstel (Gerstel, Mulheim a/d Ruhr, Germany). This is a post-run macro that expands the scope of the function of the Agilent ChemStation software, which allows the 3-dimensional raw data supplied by the mass spectrometry (retention time, *m*/*z* fragmentation and intensities) to be reduced to 2-dimensional data that can then be properly used by statistical software for further elaboration. The intensities of a sample are added as a function of the masses. The result is a data matrix of 91,980 data in which the rows report the samples and the columns report the intensity assigned to each mass.

Chemometric analyses were carried out using Pirouette software ver. 4.5 (Infometrix, Inc., Bothell, WA, USA). Principal component analysis (PCA), Partial Least Square Discriminant Analysis (PLS-DA) and Partial Least Square (PLS) regression were used. Heat map visualisation, One-way ANOVA and t-tests were performed on the sensorial results using XLSTAT (Addinsoft, New York, NY, USA).

## 4. Conclusions

The results show that the combination of HS-SPME-MS fingerprints and chemometrics is a promising technique for use as a TAS system working as a high throughput solution for the prediction of the in-cup coffee sensory quality of incoming coffee beans. Sensory quality control and evaluation is crucial if the coffee industry is to satisfy the ever-increasing demand for coffee with specific sensory attributes. The described methods would allow trained panellists to be exempted (at least partially) from routine tasting and focus their activity on new products and sensory attributes. The ambitious challenge of this study was based on the exploration of the ability of this analytical approach to predict in-cup coffee quality, including representative coffee samples of different origins, species and postharvest treatments, as occurs in industry quality control upon the acceptance of incoming beans. The study has demonstrated that this approach and the use of a multi-note model to predict global coffee sensory profiles requires a number of compromises, in terms of model robustness and acceptance of the errors in prediction. The high errors in prediction, in particular for samples with high sensory scores, probably occurred because the number of high scored samples was lower than that of the low scored samples, making this part of the score range less well represented in the sample sets. A second explanation may involve the sensory scores measured by the panel; high scores are more difficult to define and require a precise alignment.

As a general consideration, the main limit of this study is the number of coffee samples, which is only a snapshot of the number of coffees that may be processed in a plant, and is therefore not sufficient to obtain fully reliable and robust models. Automatic screening to predict the cup-quality of the raw material requires a huge repository of sensory and instrumental data. Furthermore, this approach operates, in terms of chemometric data processing, within an order of magnitude of hundreds of samples with similar qualities, as shown by Ribeiro on fifty three Arabica coffees and Lindinger on 18 espresso coffees (Ristretto and Lungo types). For higher numbers of samples, other data mining approaches should be considered in the development of the prediction tool, e.g., artificial neural networks and deep learning algorithms [23,41,43].

## Figures and Tables

**Table 1:** Descriptive statistics for the sensory attributes of coffee samples.

Attributes	Mean	S.D.	Minimum	Maximum	CV
**Acid**	1.79	1.68	0.10	7.80	0.93
**Bitter**	1.50	1.70	0.20	9.00	1.14
**Aromatic Intensity**	6.71	1.29	1.00	10.00	0.19
**Flowery**	1.08	1.76	0.00	9.00	1.62
**Fruity**	0.69	1.49	0.00	10.00	2.16
**Nutty**	1.41	2.06	0.00	9.00	1.46
**Woody**	1.36	2.02	0.00	8.00	1.49
**Spicy**	0.76	1.57	0.00	8.00	2.07
**Overall Quality**	6.63	1.46	0.60	10.00	0.22

**Table 2:** Significant ions selected from the partial least square discriminant analysis (PLS-DA) by variable importance for projection (VIP) for each sensory attribute.

Flowery			Fruity			Acid			Bitter			Nutty			Spicy			Woody			Aromatic Intensity			Overall Quality		
*m/z*	VIP	SD	*m/z*	VIP	SD	*m/z*	VIP	SD	*m/z*	VIP	SD	*m/z*	VIP	SD	*m/z*	VIP	SD	*m/z*	VIP	SD	*m/z*	VIP	SD	*m/z*	VIP	SD
37	2.012	0.201	42	2.060	0.197	36	1.822	0.208	37	1.751	0.171	42	2.668	0.795	96	1.839	0.175	110	1.626	0.085	100	1.819	0.262	79	2.060	0.316
42	1.967	0.184	45	1.999	0.176	37	1.814	0.201	42	1.722	0.170	61	2.266	0.557	36	1.830	0.182	36	1.612	0.114	36	1.717	0.096	37	2.054	0.265
45	1.960	0.213	46	1.887	0.188	38	1.775	0.168	45	1.714	0.180	64	2.218	0.542	37	1.824	0.189	37	1.603	0.108	37	1.717	0.118	38	1.925	0.220
56	1.953	0.196	52	1.884	0.183	45	1.769	0.212	56	1.682	0.140	66	2.003	0.751	38	1.819	0.168	42	1.602	0.109	38	1.697	0.106	39	1.858	0.296
57	1.821	0.157	54	1.877	0.304	46	1.752	0.190	57	1.667	0.079	67	1.955	0.408	45	1.800	0.144	45	1.564	0.103	39	1.666	0.113	40	1.816	0.140
59	1.795	0.349	56	1.847	0.138	56	1.703	0.211	60	1.666	0.074	72	1.954	0.452	46	1.699	0.123	56	1.549	0.109	40	1.664	0.198	41	1.781	0.258
60	1.757	0.133	57	1.826	0.172	57	1.675	0.176	62	1.659	0.103	80	1.890	0.478	50	1.681	0.224	57	1.546	0.036	41	1.664	0.269	42	1.729	0.079
61	1.755	0.139	58	1.805	0.264	60	1.655	0.165	63	1.642	0.056	93	1.868	0.480	53	1.645	0.054	60	1.529	0.049	45	1.651	0.208	44	1.696	0.255
62	1.748	0.127	60	1.787	0.197	62	1.621	0.153	64	1.632	0.088	94	1.810	0.396	55	1.609	0.074	62	1.514	0.038	48	1.634	0.164	50	1.671	0.409
63	1.734	0.113	61	1.775	0.252	63	1.610	0.141	65	1.594	0.050	95	1.693	0.527	56	1.609	0.203	63	1.512	0.074	50	1.592	0.265	51	1.655	0.370
64	1.732	0.097	62	1.754	0.184	64	1.609	0.114	66	1.591	0.185	96	1.648	0.667	57	1.575	0.180	64	1.501	0.047	51	1.584	0.130	52	1.644	0.346
65	1.716	0.169	63	1.750	0.213	65	1.608	0.178	67	1.589	0.071	100	1.646	0.674	60	1.572	0.126	65	1.497	0.053	52	1.580	0.197	53	1.641	0.286
66	1.709	0.119	64	1.731	0.129	66	1.608	0.154	74	1.580	0.045	106	1.638	0.764	61	1.532	0.166	66	1.491	0.056	53	1.548	0.145	54	1.607	0.388
67	1.688	0.261	65	1.723	0.121	67	1.597	0.132	75	1.579	0.096	107	1.629	0.609	63	1.531	0.098	67	1.489	0.047	55	1.544	0.104	55	1.596	0.279
69	1.687	0.174	66	1.697	0.315	72	1.565	0.067	76	1.564	0.206	108	1.612	0.471	64	1.530	0.067	69	1.480	0.095	57	1.525	0.152	58	1.571	0.383
72	1.673	0.173	67	1.654	0.294	74	1.557	0.198	77	1.561	0.030	109	1.567	0.493	65	1.503	0.057	72	1.480	0.075	59	1.522	0.274	59	1.562	0.092
74	1.643	0.232	74	1.647	0.279	76	1.524	0.129	78	1.534	0.107	110	1.438	0.586	66	1.497	0.160	74	1.477	0.097	60	1.517	0.194	61	1.559	0.388
75	1.612	0.291	75	1.621	0.144	77	1.511	0.097	80	1.504	0.039	114	1.429	0.719	68	1.497	0.180	75	1.471	0.068	61	1.503	0.129	67	1.551	0.223
77	1.585	0.222	76	1.618	0.153	78	1.492	0.129	89	1.477	0.076	121	1.418	0.711	69	1.493	0.075	76	1.461	0.068	68	1.492	0.197	68	1.548	0.327
78	1.578	0.259	77	1.609	0.203	79	1.492	0.101	92	1.451	0.093	122	1.382	0.613	70	1.488	0.219	77	1.451	0.077	69	1.481	0.178	69	1.533	0.253
80	1.559	0.349	78	1.585	0.146	80	1.470	0.153	93	1.423	0.061	135	1.379	0.693	72	1.478	0.119	78	1.446	0.105	70	1.468	0.230	70	1.497	0.190
89	1.512	0.415	79	1.580	0.199	91	1.464	0.143	94	1.417	0.063	136	1.357	0.494	74	1.464	0.203	80	1.435	0.101	72	1.463	0.220	71	1.478	0.128
92	1.495	0.475	80	1.565	0.344	92	1.462	0.125	95	1.417	0.049	159	1.356	0.614	75	1.445	0.250	89	1.430	0.118	73	1.462	0.190	72	1.477	0.520
93	1.487	0.285	92	1.554	0.344	93	1.435	0.096	96	1.412	0.230	160	1.327	0.645	77	1.444	0.130	91	1.425	0.110	74	1.457	0.310	75	1.450	0.107
94	1.480	0.186	93	1.487	0.365	94	1.399	0.157	100	1.399	0.074				78	1.440	0.113	92	1.419	0.115	79	1.450	0.112	78	1.416	0.363
95	1.468	0.172	94	1.435	0.252	95	1.392	0.140	104	1.394	0.063				80	1.436	0.165	93	1.418	0.122	81	1.435	0.190	80	1.409	0.433
96	1.445	0.439	95	1.372	0.383	96	1.388	0.195	105	1.392	0.045				89	1.425	0.181	94	1.417	0.045	82	1.421	0.359	81	1.386	0.436
100	1.423	0.137	96	1.366	0.237	100	1.376	0.127	106	1.374	0.075				91	1.422	0.277	95	1.405	0.108	83	1.421	0.246	82	1.352	0.198
106	1.419	0.286	103	1.354	0.330	104	1.374	0.300	107	1.366	0.116				92	1.403	0.138	96	1.401	0.157	86	1.415	0.136	83	1.340	0.291
107	1.412	0.302	104	1.316	0.205	105	1.374	0.247	108	1.362	0.162				93	1.384	0.190	100	1.396	0.059	87	1.401	0.222	86	1.338	0.405
108	1.404	0.242	105	1.287	0.170	106	1.368	0.086	109	1.350	0.225				94	1.344	0.146	104	1.392	0.126	91	1.375	0.265	87	1.317	0.297
109	1.389	0.275	106	1.285	0.182	107	1.367	0.125	110	1.347	0.114				95	1.338	0.367	105	1.378	0.072	92	1.343	0.258	88	1.295	0.280
110	1.328	0.158	107	1.254	0.158	108	1.366	0.241	117	1.340	0.129				97	1.320	0.351	106	1.369	0.066	95	1.310	0.216	94	1.277	0.272
112	1.307	0.322	108	1.253	0.197	109	1.365	0.163	118	1.339	0.204				98	1.312	0.151	107	1.351	0.103	96	1.280	0.333	97	1.272	0.338
118	1.274	0.259	109	1.235	0.438	110	1.345	0.101	119	1.324	0.070				100	1.298	0.185	108	1.329	0.166	97	1.267	0.319	98	1.271	0.265
119	1.239	0.301	110	1.228	0.315	115	1.335	0.174	120	1.309	0.120				103	1.267	0.209	109	1.327	0.085	98	1.255	0.134	99	1.251	0.228
120	1.211	0.231	113	1.223	0.272	117	1.329	0.109	121	1.291	0.165				104	1.260	0.425	112	1.303	0.099	99	1.214	0.393	111	1.213	0.446
121	1.202	0.215	117	1.221	0.308	118	1.318	0.150	122	1.284	0.120				105	1.254	0.371	113	1.285	0.088	109	1.210	0.424	112	1.206	0.226
122	1.186	0.459	118	1.220	0.228	119	1.309	0.263	123	1.283	0.120				106	1.219	0.136	115	1.285	0.175	110	1.185	0.510	113	1.190	0.333
123	1.176	0.525	119	1.206	0.375	120	1.292	0.207	124	1.276	0.125				107	1.209	0.231	116	1.258	0.144	111	1.133	0.324	123	1.187	0.382
124	1.150	0.193	120	1.187	0.329	121	1.288	0.164	125	1.274	0.081				108	1.180	0.122	117	1.255	0.153	112	1.131	0.483	125	1.143	0.411
126	1.112	0.242	121	1.180	0.249	122	1.268	0.196	131	1.265	0.137				109	1.173	0.196	118	1.244	0.121	116	1.109	0.305	126	1.120	0.353
134	1.100	0.479	122	1.167	0.391	123	1.265	0.186	132	1.263	0.195				110	1.168	0.209	119	1.230	0.179	126	1.055	0.445	138	1.096	0.196
135	1.093	0.381	123	1.156	0.277	124	1.225	0.167	134	1.259	0.141				112	1.148	0.203	120	1.224	0.128	140	1.036	0.459	139	1.068	0.285
136	1.085	0.304	124	1.146	0.348	125	1.215	0.186	135	1.231	0.118				115	1.146	0.138	121	1.223	0.125	141	1.025	0.370	140	1.063	0.585
137	1.072	0.387	125	1.145	0.401	129	1.195	0.207	136	1.175	0.145				117	1.142	0.142	122	1.214	0.232	166	1.014	0.527	141	1.060	0.184
139	1.053	0.336	132	1.132	0.187	134	1.173	0.166	137	1.174	0.160				118	1.131	0.188	123	1.206	0.130				161	1.028	0.269
146	1.044	0.371	134	1.102	0.327	135	1.139	0.155	145	1.160	0.137				119	1.124	0.224	124	1.205	0.111						
147	1.040	0.379	135	1.101	0.322	136	1.138	0.241	146	1.157	0.176				120	1.108	0.210	125	1.188	0.156						
150	1.021	0.471	136	1.099	0.220	137	1.125	0.411	150	1.138	0.122				121	1.102	0.257	127	1.177	0.157						
160	1.018	0.521	137	1.097	0.306	145	1.108	0.348	151	1.137	0.271				122	1.098	0.253	131	1.176	0.106						
164	1.002	0.467	145	1.092	0.248	146	1.096	0.246	152	1.126	0.146				123	1.085	0.199	132	1.168	0.190						
			146	1.074	0.213	148	1.092	0.371	164	1.112	0.214				124	1.084	0.296	134	1.145	0.166						
			150	1.039	0.274	150	1.077	0.204							126	1.056	0.303	135	1.131	0.146						
			152	1.006	0.280	151	1.046	0.225							132	1.037	0.488	136	1.119	0.226						
			164	1.001	0.272	152	1.031	0.405							134	1.036	0.420	137	1.114	0.208						
						160	1.028	0.231							135	1.034	0.318	139	1.107	0.152						
						164	1.011	0.142							136	1.023	0.331	146	1.092	0.169						
															137	1.016	0.487	148	1.089	0.250						
															145	1.015	0.500	150	1.086	0.218						
															150	1.015	0.383	151	1.082	0.300						
															151	1.010	0.214	152	1.017	0.183						
															164	1.001	0.384	160	1.014	0.263						
																		164	1.003	0.129						
**Total number**																										
52			56			58			53			24			63			64			46			47		

**Table 3:** Multi-note model performance summary.

	Single-Note Model Performance				Multi-Note Model Performance			
*Sensory Note*	*Model Factors*	*R2val*	*RMSEV*	*RMSEP*	*Model Factors*	*R2val*	*RMSEV*	*RMSEP*
*Acid*	3	0.663	1.129	0.946	3	0.856	0.726	1.192
*Bitter*	4	0.817	1.142	1.063	4	0.936	0.626	1.315
*Woody*	4	0.669	1.570	1.725	4	0.884	1.003	2.306
*Flowery*	4	0.746	1.038	1.345	4	0.907	0.651	1.964
*Fruity*	4	0.661	1.026	1.499	2	0.790	0.785	1.598
*Spicy*	1	0.792	0.963	1.209	3	0.784	0.977	1.194
*Nutty*	6	0.544	1.506	1.661	4	0.893	0.864	1.891
*Aroma intensity*	1	0.557	0.936	1.296	4	0.764	0.627	1.642
*Overall quality*	4	0.556	0.936	2.120	4	0.756	0.726	2.239

## Supplementary Materials

The following is present in supplementary material available online, Figure S1: PCA scores plots of coffee samples.
