# Supplementary figures and images for: HS-SPME-MS-Enose Coupled with Chemometrics as an Analytical Decision Maker to Predict In-Cup Coffee Sensory Quality in Routine Controls: Possibilities and Limits
Source: Molecules. 2019 Dec 10;24(24):4515. doi: 10.3390/molecules24244515 (PMC6943652; doi:10.3390/molecules24244515)

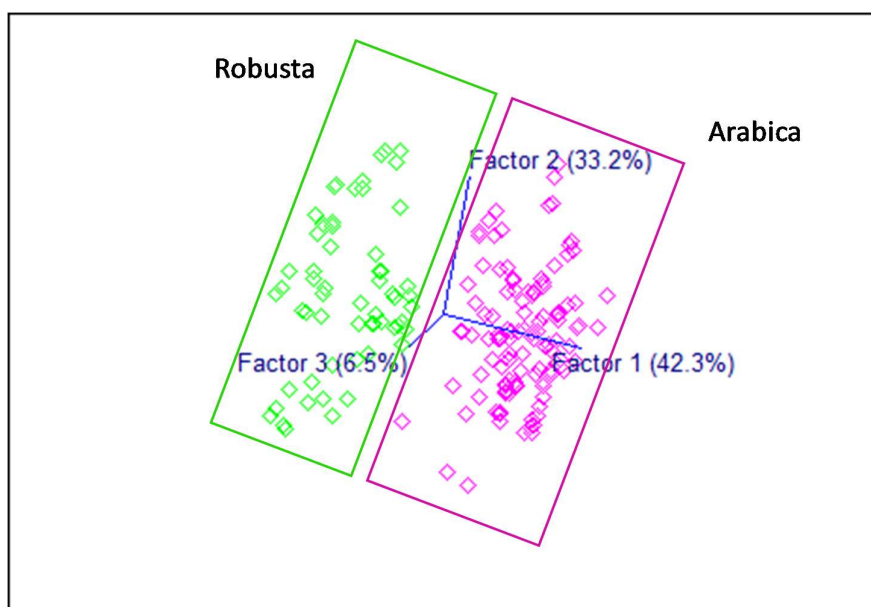

Figure S1. PCA scores plot of the coffee samples. Pre-processing: Pareto scaling

Supplement: Supplementary file 1 [file molecules-24-04515-s001.pdf]
